# Machine learning-based identification and rule-based normalization of adverse drug reactions in drug labels

**DOI:** 10.1186/s12859-019-3195-5

**Published:** 2019-12-23

**Authors:** Mert Tiftikci, Arzucan Özgür, Yongqun He, Junguk Hur

**Affiliations:** 1 Department of Computer Engineering, Boğaziçi University, İstanbul, 34342 Turkey; 2 Unit for Laboratory Animal Medicine, Department of Microbiology and Immunology, Center for Computational Medicine and Bioinformatics, University of Michigan Medical School, Ann Arbor, 48109 MI USA; 3 Department of Biomedical Sciences, University of North Dakota School of Medicine and Health Sciences, 1301 North Columbia Rd, Grand Forks, North Dakota 58202 USA

**Keywords:** Text mining, Entity recognition, Entity normalization, Adverse drug reaction, Deep learning, Machine learning

## Abstract

**Background:**

Use of medication can cause adverse drug reactions (ADRs), unwanted or unexpected events, which are a major safety concern. Drug labels, or prescribing information or package inserts, describe ADRs. Therefore, systematically identifying ADR information from drug labels is critical in multiple aspects; however, this task is challenging due to the nature of the natural language of drug labels.

**Results:**

In this paper, we present a machine learning- and rule-based system for the identification of ADR entity mentions in the text of drug labels and their normalization through the Medical Dictionary for Regulatory Activities (MedDRA) dictionary. The machine learning approach is based on a recently proposed deep learning architecture, which integrates bi-directional Long Short-Term Memory (Bi-LSTM), Convolutional Neural Network (CNN), and Conditional Random Fields (CRF) for entity recognition. The rule-based approach, used for normalizing the identified ADR mentions to MedDRA terms, is based on an extension of our in-house text-mining system, SciMiner. We evaluated our system on the Text Analysis Conference (TAC) Adverse Drug Reaction 2017 challenge test data set, consisting of 200 manually curated US FDA drug labels. Our ML-based system achieved 77.0% F1 score on the task of ADR mention recognition and 82.6% micro-averaged F1 score on the task of ADR normalization, while rule-based system achieved 67.4 and 77.6% F1 scores, respectively.

**Conclusion:**

Our study demonstrates that a system composed of a deep learning architecture for entity recognition and a rule-based model for entity normalization is a promising approach for ADR extraction from drug labels.

## Background

Pharmacovigilance is defined as “the science and activities relating to the detection, assessment, understanding and prevention of adverse effects or any other drug problem” [1]. It is impossible to know all possible adverse events of a particular drug, since generalizability of the clinical trials are low, sample sizes are small, and duration is short. FDA uses the Adverse Event Reporting System (FAERS) to detect adverse events. FAERS includes mandatory reports from pharmaceutical companies and reports that have been submitted to MedWatch directly. ADRs are still in the top 10 leading causes of death and cost approximately $75 billion annually in the United States [2].

In addition to using medical reports for detecting ADRs [3], it has been proposed to use data from social media [4], since users tend to discuss their sicknesses, treatments and prescribed drugs and their effects in social media platforms. These discussions are not only confined to social networks specifically dedicated to health-related issues, but they also exist in generic platforms which could all be used for multi-corpus training to increase the accuracy of text mining systems for ADR recognition [5].

The current approach for FAERS case report review requires manual reading of the text of the drug labels in order to determine whether a candidate ADR has been reported before or not. The automation of the extraction of the ADRs from drug labels would increase the efficiency of this process. Preparing a lexicon [4] for detection of ADRs requires a lot of manual work and also limits a system’s effectiveness to the extent of the lexicon. Syntactic and semantic patterns have been used in order to remedy the shortcomings of lexicon-based approaches [6]. Detailed information on ADR extraction with different techniques on various data sources is available in [7, 8].

Recently, the Text Analysis Conference (TAC), a series of workshops organized to encourage research in Natural Language Processing and related applications (https://tac.nist.gov/2017/), included a special track focused on adverse drug reaction (ADR) extraction from drug labels. The TAC-ADR 2017 challenge targeted the automatic extraction of ADR mentions from drug labels and normalization of them through MedDRA. A mention of an entity can be defined as the portion of a text that corresponds to a certain entity such as an ADR. For example, given the sentence “Exclusive of an uncommon, mild injection site reaction, no adverse reactions to ^11^C-choline have been reported.” obtained from the drug label of choline, “injection site reaction” is an ADR mention and “mild” is a severity mention.

Using a comprehensive and well-structured dictionary is critical in literature mining-based application. For ADR, Medical Dictionary for Regulatory Activities (MedDRA) terms [9], SNOMED CT [10] as well as a few biomedical ontologies developed by us such as Ontology of Adverse Events (OAE) [11] and Ontology of Drug Neuropathy Adverse Events (ODNAE) [12] can be used. The most widely-used dictionary for supporting ADR reporting is MedDRA, which is a clinically validated standardized medical terminology dictionary (and thesaurus), consisting of five levels of hierarchy [9].

The MedDRA dictionary organizes various ADRs using a five-level hierarchy. The bottom layer is Lowest Level Terms (LLT) at the bottom, followed by Preferred Terms (PT), High Level Terms (HLT), High Level Group Terms (HLGT), and System Organ Class (SOC). While individual ADR cases are usually coded for data entry at the most specific LLT level, the outputs of counts or cases are usually provided at the PT level. The term “Injection site reaction” in the sentence above is an HLT term MedDRA, which has a MedDRA ID “10022095”. Under this term, there are many PTs and LLTs.

In this study, we investigated the integration of machine learning and dictionary/rule-based methods in identifying ADR terms from drug labels and normalizing them to MedDRA preferred terms (PT). Our best results were achieved by an integrated system that is based on a deep learning model for entity mention extraction and a dictionary/rule-based SciMiner method for the normalization of the extracted ADRs to MedDRA terms. Our methods and results are described in the following sections.

## Results

The current study focused on extracting the mentions from a given drug label and normalizing them to appropriate MedDRA PTs. The deep model worked at the sentence level of the texts; therefore, the texts had to be split to the sentence level first as the initial process.

The NLTK tokenizer [13] was used to identify the tokens in the sentences and transformed every drug label file into the CoNLL format. The sentences were separated by an empty line and every token was written on a separate line. An example sentence is shown in Table 1 and its CoNLL format is shown in Table 2, where every line consists of 6 columns and starts with the token itself. The second column holds the tag type of the token, which was encoded with BIO2 [14] chunking representation. “B” denotes that the token is the beginning of an entity mention, “I” denotes that the token is inside of a mention, and “O” (Outside) indicates that the token is not part of a mention. For example, the tags of an ADR term “hypersensitivity reactions” are “B-ADR I-ADR” according to this representation. The following columns show the location of the token within a label. The first one of those is the id of the section. The second one is the start position of the token within the section and the last one shows the length of the token.

**Table 1:** Example sentence from drug label and its representation in XML format. The text drug label data were provided in XML format and this figure illustrates an example sentence exerted from drug label “Choline”. These XML-formatted labels from the TAC include three main sections: “Raw Text” containing the original texts from ADR-relevant sections from drug labels; “Related Mentions” containing the manually curated ADRs; and “Related Reactions” containing normalized ADRs in terms of MedDRA terms

Raw Text	Long-term cumulative radiation exposure is associated with an increased risk for cancer.
Related	<Mention id="M10" section="S2" type="Factor" start="2309" len="4" str="risk" /> <Mention id="M11" section="S2" type="AdverseReaction" start="2318" len="6" str="cancer" />
Mentions	<Reaction id="R4" str="cancer"> <Normalization id="R4.N1" meddra_pt="Neoplasm malignant" meddra_pt_id="10028997" meddra_llt="Cancer" meddra_llt_id="10007050" /> </Reaction>

**Table 2:** BIO sentence processing example. This table illustrates a BIO (beginning-inside-outside) processing of a sentence, obtained from a drug label of “Zylelig”, an anti-cancer medicine. Every drug sectioned with a unique id (S3 in the given sentence). Every token within the sections has the property Offset which is the character count before the first character of a given token

Raw Text	BIO encoding	Section	Offset	Length
Fatal	B-ADR	S3	2763	5
and	O	S3	2769	3
serious	B-SEV	S3	2773	7
intestinal	B-ADR	S3	2781	10
perforation	I-ADR	S3	2792	11
occurred	O	S3	2804	8
in	O	S3	2813	2
Zydelig-treated	O	S3	2816	15
patients	O	S3	2832	8
.	O	S3	2840	1

### Named entity recognition (NER) data processing

For the present study, two different approaches were employed in terms of named entity recognition and ADR normalization as summarized in Table 3. Briefly, for NER, the Set#1 used the machine learning-based method alone, Set#2 used the rule- and dictionary-based SciMiner method alone. Normalization of the ADRs that were identified by ML-approach was done by SciMiner using dictionary- and rule-based approach. We have developed pipelines for both methods and performance of these approaches is summarized below.

**Table 3:** Summary of approaches

Set	Named Entity Recognition (NER) Method	ADR Normalization
Set#1	ML	SciMiner
Set#2	SciMiner	SciMiner

### MedDRA ADR normalization

**Fig. 1:**
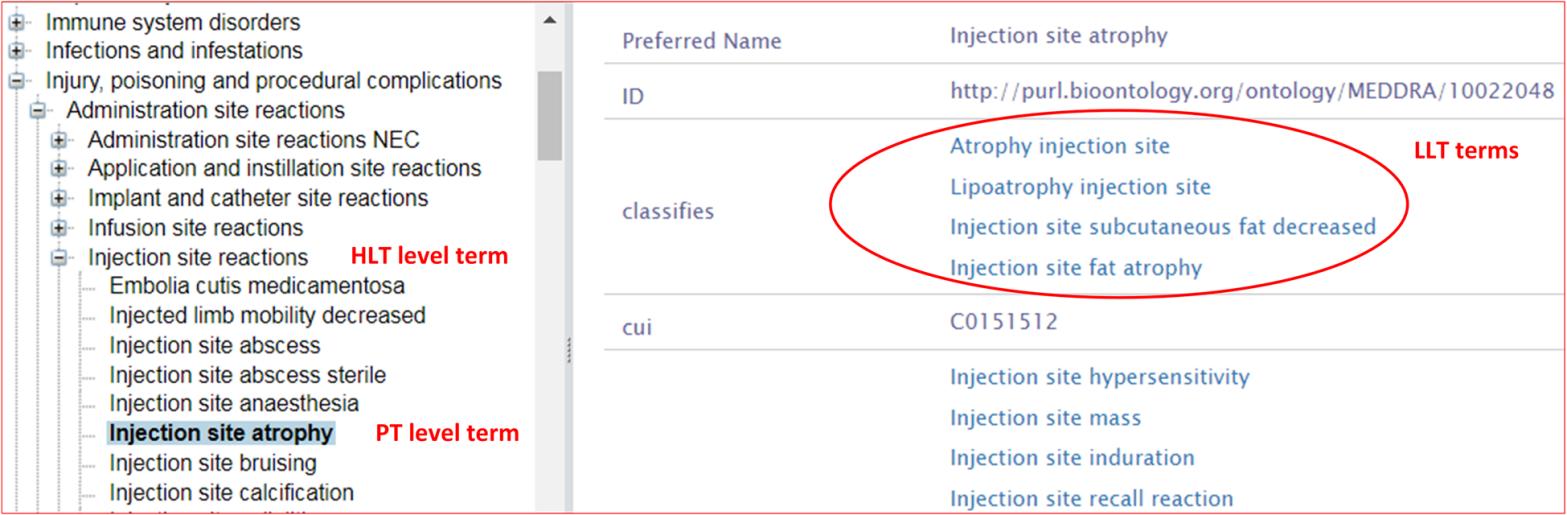
Illustration of MedDRA PT and LLT hierarchy with example. A single medical concept is represented by a PT which could be described with many different ways that could have different lexical variants and synonyms. With the help of LLTs, it is pretty beneficial to classify a given text to one of PTs

In our study, the PT-layer terms of MedDRA were used as the dictionary of ADRs. As shown in Fig. 1, the ‘injection site atrophy’ is a MedDRA PT, and it has many associated LLTs such as ‘atrophy inject site’, and ‘injection site fat atrophy’. These LLTs are synonyms or subclasses of their corresponding PTs. The MedDRA information was preprocessed and loaded to the SciMiner system. The identified ADR terms were first mapped to any LLTs and PTs. ADRs mapped to LLTs were then further normalized to their corresponding PTs.

### TAC contest performance evaluation result

We participated in the task of the TAC-ADR 2017 challenge with an aim for automatic extraction of ADR mentions through MedDRA. There are 2309 labels exists in the TAC dataset from which 200 of them have been annotated. Participants received only 101 annotated labels and extracted mentions from rest of the 2208 labels without knowing which 99 labels were included in the test set (see more details in the Drug Label Dataset section). Our performance record is shown in Table 4. Briefly, these two sets accomplished overall F1-measures of 77.0 and 63.4% in NER identification, and micro-level F1-measures of 82.6 and 77.6% and macro-level F1-measures of 80.5 and 75.6% in normalizing to appropriate MedDRA PT, respectively. The best performance was achieved when NERs were identified using our ML-based approach and then normalized to MedDRA Preferred Terms by dictionary- and rule-based approach (SciMiner). Our top performing result was ranked at 4th among the 12 results competed for the normalization performance in the 2017 TAC-ADR track [15].

**Table 4:** Performance evaluation results. Results are given in percentages (%)

			Set#1	Set#2
Identification		Precision	76.5	65.5
		Recall	77.5	61.4
		F1 score	77.0	63.4
Normalization	micro	Precision	88.8	74.6
		Recall	77.2	81.0
		F1 score	82.6	77.6
	macro	Precision	88.2	73.1
		Recall	75.8	79.9
		F1 score	80.5	75.6

## Discussion

There were many teams participated in the TAC 2017 shared task of adverse reaction extraction. Our model closely resembles the best performing models for Task 1 from [16, 17] since we all used the Bi-LSTM as the core of the sequence tagger. Best-performing team, with the F1 score of 82.48%, used a cascading Bi-LSTM model for extraction ADRs. They have trained two Bi-LSTMs, while the first one only tags ADR mentions, the second one tags the other mention types that are related to a single chosen ADR mention. This model is expected to perform better, since the mentions other than ADRs were not annotated when they were not related to an ADR mention.

Training a single tagger for all entity types become less efficient as our model and model of [17] do. Even though they [16] used BIO tagging, which is not fit to handle overlapping and disjoint entities their model performed well, because they combined disjoint entities during tagging. This approach allowed all mention chunks to be continuous, thus making BIO tags to be more consistent. They developed rules that are learned from the training set for later generate disjoint entities that have tagged as the output of the trained model. The major difference between our model and the second-best performing model of [17], with the F1 score of 76.97%, probably is the BIOHD tagging scheme. This scheme specifically developed to handle disjoint and overlapping entities with the addition of new labels for each condition. They also trained a second sub-model only to classify given a disjoint entity pair to be merged or not.

In the normalization of the extracted ADR mentions onto the MedDRA ontology, the best performing team was again [16] with a micro-F1 score of 86.91% and a macro-F1 score of 85.33%. It is hard to compare different approaches to this problem since this task is dependent on the performance of the first one. Performance levels could be said to be roughly close with us favored since the difference between ADR extraction performance is 6.2% between their model and ours whereas the difference in the micro-F1 score is 4.33% and in the macro-F1 score is 4.83%.

As future work, we will investigate incorporating ontology and dictionary knowledge into the deep learning model. Also updating the word embeddings [18], making an extensive parameter search and solving the problems with preprocessing are likely to increase the performance of the deep learning model. Using a more suitable tagging scheme that could handle irregular entities would enable the machine learning algorithms to be more efficient.

## Conclusions

In this study, we employed two different methods for detecting mentions of type ADR, drug class, animal, severity, factor, and negations from drug labels. The neural network-based approach outperformed the dictionary- and rule-based approach in terms of extracting ADRs. Our study suggests that a system composed of a deep learning architecture for entity recognition and a rule-based model for entity normalization is a promising approach for ADR extraction from drug labels.

## Methods

**Fig. 2:**
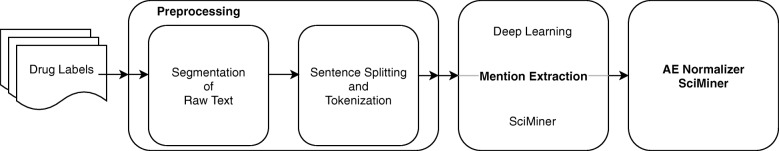
Overall workflow. This figure illustrates our overall workflow in the present study. Drug labels included in the TAC dataset were analyzed to identify ADRs and normalized them through MedDRA v20. Pre-processing was needed only when the deep learning architecture was used

A high-level description of our integrated deep learning and dictionary/rule-based approach for entity detection and normalization is illustrated in Fig. 2. We investigated the performance of using both a machine learning approach and a dictionary/rule-based approach for mention-extraction task of the TAC-ADR 2017 challenge, whose goal was to extract entity mentions in drug labels such as ADR, drug class, animal, severity, factor, and negation. For example, in the sample sentence provided in the Introduction section, the severity mention “mild” has been annotated, since it defines the severity of the ADR “injection site reaction”. If “mild” occurs in a drug label in another context such as the symptoms of a disease being mild, then it is not annotated, since it is not related to an ADR.

Another main task in this TAC-ADR challenge was to properly normalize the positive ADRs detected in the previous task to their corresponding MedDRA terms. For ADR normalization we extended and used our in-house literature mining program SciMiner [19], which is a dictionary- and rule-based literature mining platform for identification of genes and proteins in a context-specific corpus. MedDRA preferred terms (PT) and lowest level terms (LLT) were added to SciMiner, which normalized the positive ADRs to MedDRA preferred terms. MedDRA has the medical terminology hierarchy arranged from very specific to very general, where LLT is the most specific layer and PT is on top of it.

The machine learning component operates on sentence level and requires the input to be tokenized. Therefore, the first step of our system was to transform the drug labels, given in XML format, to sentence-split and tokenized format. The NLTK package (http://www.nltk.org) was used for sentence splitting and tokenization. Since the documents were not well formatted and contained tables, a Python script was internally prepared to detect text pieces and table parts. These initial preprocessing operations increased the performance of the sentence splitter. The machine learning and dictionary-based components of the system are described in more detail in the following subsections.

### Neural network architecture

A deep learning model designed for extracting named entity recognition (NER), which makes use of bi-directional Long Short-Term Memory (Bi-LSTM), Convolutional Neural Network (CNN), and Conditional Random Fields (CRF) [20], was used for the extraction of ADR mentions. We used the implementation proposed by [21] which has minor differences from [20]. In the paper [21], the authors focused on the parameter tuning of neural networks on some tasks including named entity recognition. We used their suggested configuration of hyper-parameters while training the model with the difference of pre-trained word embeddings and maximum epoch count in training. The model works on the sentence level, where every token is represented by a vector. Here, we describe the network starting from the creation of the input vectors to the prediction of the entity tags, which are calculated for every token of a given sentence.

### Combined word Embeddings

**Fig. 3:**
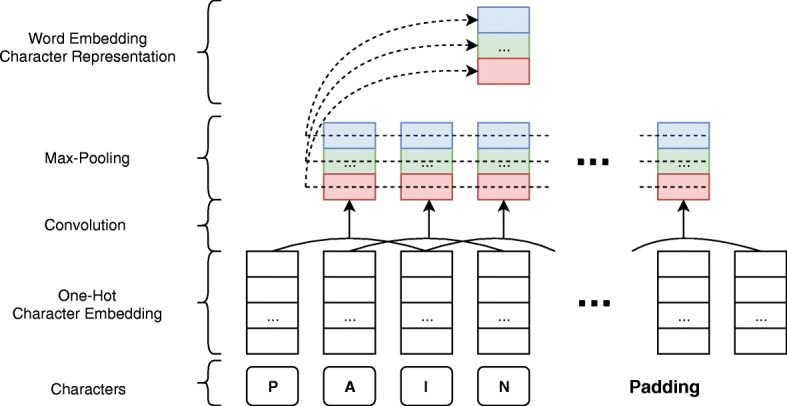
Character representation generation with CNN. This component can only work on the same sized inputs; therefore, inputs are extended with paddings which are inconsequential in the feature extraction. Also, this representation only shows calculation of one filter on the Convolution part, but CNN used in the model have 30 filters

Every token in a given sentence was transformed into a vector before being fed into the model. These vectors consist of three parts, namely character embeddings, word embeddings, and case embeddings. The character embeddings were generated by a convolutional neural network (CNN) that runs over the characters of a given token. This representation has been shown to be powerful in encoding morphological information [20], which we expect to be useful in the biochemical domain as well. At the first step, the tokens were transformed into their matrix representation by concatenating their character embeddings. Since CNNs work on fixed length input, all matrices were filled with padding to the length of the longest word in the vocabulary. Filter size was set to be 3 with a stride value of 1. In total 30 filters with these parameters were used for each input token in the CNN architecture. After using a max-pooling operation, a vector of length 30 was generated for each token. Figure 3 illustrates the workflow of the generation of character embeddings using the CNN component.

The word embeddings were generated by the Word2Vec tool [22] in order to incorporate semantic information of words, since these representations had been shown to be effective in capturing semantic meanings [22]. The performance is expected to increase when these embeddings are generated from a corpus that is more related to the task; therefore, we used pre-trained embeddings that were generated using PubMed as the training corpus [23]. These vectors of length 200 were appended to the character embeddings created by CNN. While looking for the vector representation of a token, our system also looked for lower cased and normalized versions in order to reduce out-of-vocabulary (OOV) words. However, it should be noted that this process decreased the number of OOV words, but we also lost the actual casing information of tokens. In order to remedy this loss, one-hot encoded case embeddings with length 8 were appended to the word embedding vectors, obtaining the combined word embedding vectors.

### The bi-LSTM and CRF component

**Fig. 4:**
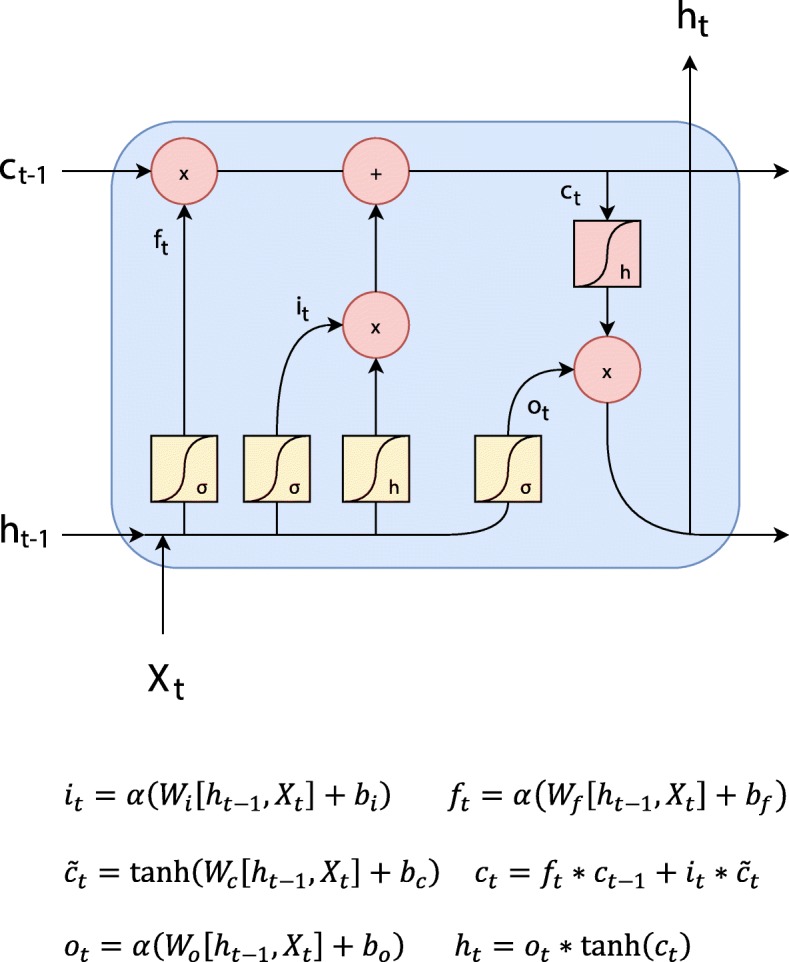
Graphical representation of LSTM module for understanding the operations. This represent a single input in a given sequence, sentence in our task. In this representation input is denoted by X which enters from below. Features that are extracted for the given input is represented by h and cell state is represented by c

Our model used a long short-term memory (LSTM) [24] component, which takes as input the combined word embeddings in order to model the context information for each word as shown in Fig. 4. LSTM is from the family of Recurrent Neural Networks (RNNs), which are designed to learn patterns within sequences [24]. Even though these components are theoretically capable of learning long distance dependencies, it is hard to train them with gradient descent due to the problems of gradient vanishing or explosion [25]. LSTMs are better in dealing with the gradient vanishing problem compared to the vanilla RNN, but they cannot solve the gradient explosion problem. As a solution to the gradient explosion problem, our model used gradient normalization [26] with the value of 1, since it has been shown to be effective in the NER task [21].

**Fig. 5:**
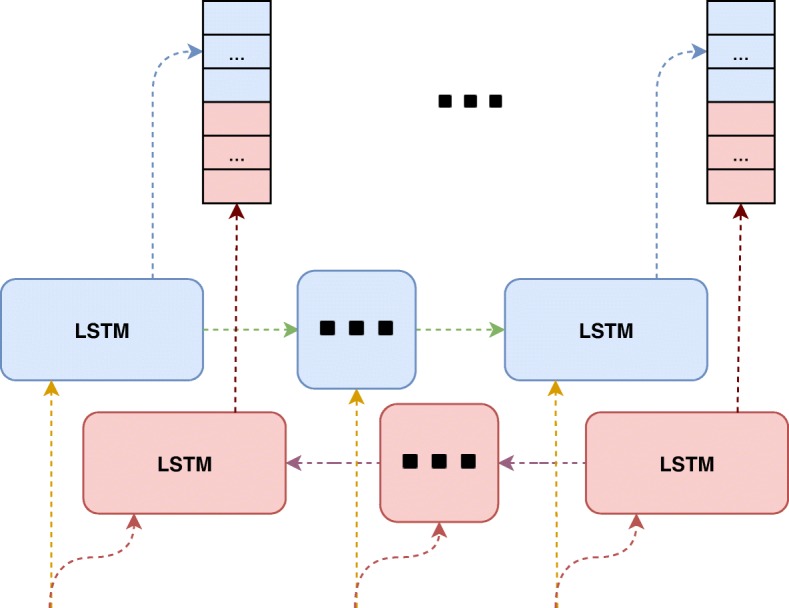
Bi-LSTM component with variational dropout (depicted by colored & dashed connections). Bi-suffix in the component name stands for the bi-directional which means there exist two identical LSTM modules running on a given input on different directions. Concatenation of extracted features of LSTMs are the output of this component. Intuition behind this is to utilize the information exist in the rest of a given sequence since single LSTM extracts latent information using only elements in the sequence before that one

For detecting NERs, it has been shown to be an effective approach to have prior knowledge about the rest of the sentence well as the beginning. Two recent studies [20, 27] used two LSTMs which run on opposite directions on the input sequences. Therefore, as shown in Fig. 5, the outputs of the two LSTMs are concatenated. Two of these Bi-LSTM components are stacked. The first Bi-LSTM has 100 recurrent units and the second one has 75 recurrent units.

Dropout [28] is a way to prevent overfitting in neural networks. However, it has been shown to be difficult to apply on RNN layers. Hence, variational dropout [29] has been applied in the Bi-LSTM layers. This method applies the same mask through time in recurrence, which is shown by colored dashed arrows in Fig. 5. Dropout of 0.25 was applied in our Bi-LSTM components.

**Fig. 6:**
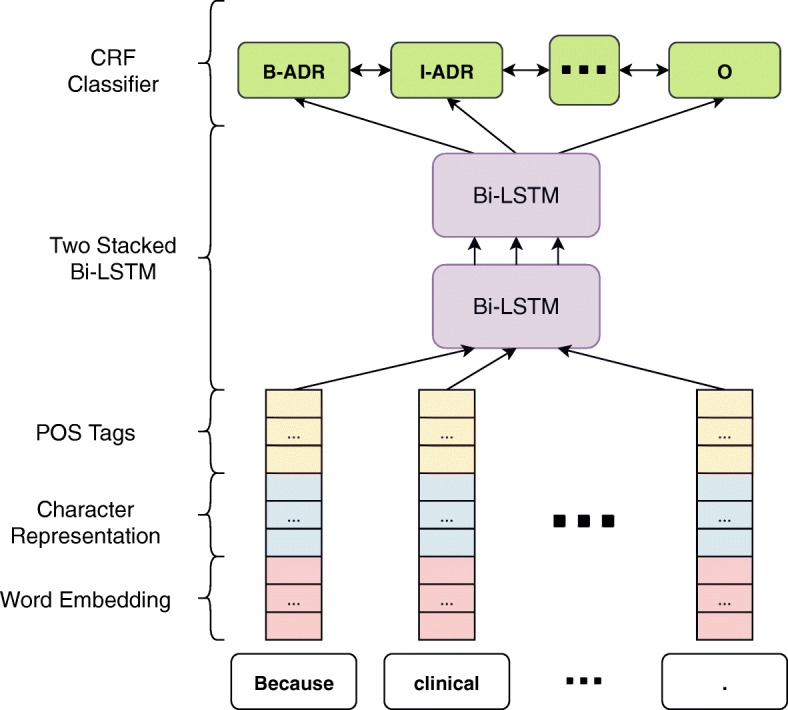
Deep learning model for NER. Complete structure of the architecture to display how described components are put together as a single named entity recognizer

The last layer is the Conditional Random Fields (CRF) [30], which does the prediction of the token tags. The TAC-ADR dataset contained non-contiguous mentions such as “Interstitial infiltration ... of the chest” with 10 words, but CRF is expected to work better if all mentions are contiguous. The CNN Bi-LSTM and CRF models are combined and used as the final deep learning model as shown in Fig. 6. The NADAM [31] optimization technique is used in the training of the combined model.

### SciMiner: dictionary- and rule-based approach

In parallel to the neural network-based approach above, we employed a dictionary- and rule-based NER approach. We used SciMiner written in Perl, which was originally developed as a web-based literature mining platform for identifying genes and proteins in biomedical literature [19]. SciMiner has been expanded to identify various biomedical ontologies such as Vaccine Ontology (VO) and Interaction Network Ontology (INO), developed by our group, resulting in specific variations of SciMiner: INO-SciMiner [32], VO-SciMiner [33], and E-coli-SciMiner [34].

We recently developed and applied an expansion of SciMiner focusing on ADR study, named as ADR-SciMiner, to a study of ontology-based literature mining and drug class effect analysis of ADRs associated with drug-induced neuropathy [35]. Manual review of these terms was also performed to identify such terms that are unlikely to be ADRs such as various cancers. Various rules for term expansion as well as exclusion to increase coverage and accuracy were implemented. For example, Perl library Lingua::EN was used to expand the base ADR dictionary allowing the inclusion of additional plural or singular forms, when only one form was included in the base dictionary. SciMiner-based approach was also used for normalizing the positive ADR terms, identified by the deep learning-based approach in the above section, to their respective MedDRA PTs.

### Drug label dataset

The TAC dataset included 200 manually curated labels (101 in the *Training* and 99 in the *Unannotated* sets) and the details have been recently published [35, 36]. These XML files contained raw texts with sections, mentions, relations and normalizations for reactions. Briefly, four annotators, including two medical doctors, one medical librarian and one biomedical informatics researcher, participated in the manual annotation process of these 200 drug labels. These annotators were all trained biomedical annotation and the drug labels were annotated independently by these annotators. Any disagreements were reconciled in pairs or collectively resolved by all four annotators. The mining performance of our approaches was evaluated using the 99 drug labels in the *Unannotated* set. The evaluation was done at the level of normalized MedDRA PTs for each drug. Recall, Precision, and F1 score were calculated.

### Irregular entity mentions

Irregular entity mentions also pose challenges for entity recognition and normalization. Irregular entity mentions can be discontinuous or overlapping. Overlapping entity mentions consist of two or more entities whose mentions overlap in the text. Discontinuous entities span text portions that are not continuous as exemplified “Angioedema of the face, lips, tongue, and/or larynx has been reported with fesoterodine.”, a sentence from the label of the drug Toviaz. The entity mentions are “Angioedema of the face”, “Angioedema of the lips”, “Angioedema of the tongue”, and “Angioedema of the larynx”. These are overlapping entities, since the text portion “Angioedema of the” is common in all four entity mentions. The last three entities are discontinuous, since there are other words between the text portion “Angioedema of the” and the remaining parts of the entity mentions. For example, in the entity mention “Angioedema of the tongue”, the words “face” and “lips” occur between the entity mention texts “Angioedema of the” and “tongue”.

## Data Availability

The original drug label text data are available through the Text Analysis Conference 2017 organizing committee.

## Abbreviations

ADR: Adverse Drug Reaction
Bi-LSTM: Bi-directional Long Short-term Memory
BIO: Begin-inside-outside
CNN: Convolutional Neural Network
CRF: Conditional Random Fields
FAERS: FDA Adverse Event Reporting System
HLGT: High Level Group Term
HLT: High Level Term
LLT: Lowest Level Term
MedDRA: Medical Dictionary for Regulatory Activities
ML: Machine Learning
NADAM: Nesterov Adaptive Moment Estimation
NER: Named Entity Recognition
NLTK: Natural Language Toolkit
OOV: Out of Vocabulary
PT: Preferred Term
RNN: Recurrent Neural Network
SOC: System Organ Class
TAC: Text Analysis Conference

## References

[1] World Health Organization and others (2002). The importance of pharmacovigilance.

[2] Ahmad SR (2003). Adverse drug event monitoring at the Food and Drug Administration. J Gen Intern Med.

[3] Gurulingappa H, Fluck J, Hofmann-Apitius M, Toldo L (2011). Identification of adverse drug event assertive sentences in medical case reports. First international workshop on knowledge discovery and health care management (KD-HCM), European conference on machine learning and principles and practice of knowledge discovery in databases (ECML PKDD).

[4] Leaman R, Wojtulewicz L, Sullivan R, Skariah A, Yang J, Gonzales G (2010). Towards Internet-age Pharmacovigilance: Extracting Adverse Drug Reactions from User Posts to Health-related Social Networks. Proceedings of the 2010 Workshop on biomedical natural language processing.

[5] Sarker A, Gonzales G (2015). Portable automatic text classification for adverse drug reaction detection via multi-corpus training. J Biomed Inform.

[6] Nikfarjam A, Gonzalez GH (2011). Pattern Mining for Extraction of mentions of adverse drug reactions from user comments. AMIA Ann Symp Proc.

[7] Harpaz R, Callahan A, Tamang S, Low Y, Odgers D, Finlayson S (2014). Text Mining for Adverse Drug Events: the Promise, Challenges, and State of the Art. Drug Safety.

[8] Karimi S, Wang C, Metke-Jimanez A, Gaire R, Paris C (2015). Text and Data Mining Techniques in Adverse Drug Reaction Detection. ACM Comput Surv.

[9] Brown EG, Wood L, Wood S (1999). The medical dictionary for regulatory activities (MedDRA). Drug Saf.

[10] Nadkarni PM, Nadkarni PM, Darer J (2010). Determining correspondences between high-frequency MedDRA concepts and SNOMED: a case study. BMC Med Inform Decis Mak.

[11] He Y, Sarntivijai S, Sarntivijai S, Lin Y, Xiang Z, Guo A (2014). OAE: the ontology of adverse events. J Biomed Semantics.

[12] Guo A, Racz R, Hur J, Lin Y, Xiang Z, Zhao L (2016). Ontology-based collection, representation and analysis of drug-associated neuropathy adverse events. J Biomed Semantics.

[13] Bird S, Loper E, Klein E (2009). Natural language processing with Python: analyzing text with the natural language toolkit.

[14] Sang EFT, Veenstra J. Representing text chunks. In: Proceedings of the ninth conference on European chapter of the Association for Computational Linguistics. Association for Computational Linguistics; 1999. p. 173–9. Available from: https://www.aclweb.org/anthology/E99-1023/.

[15] Roberts K, Demner-Fushman D, Tonning JM. Overview of the TAC 2017 Adverse Reaction Extraction from Drug Labels Track. In: Proceedings of the 2017 Text Analysis Conference. NIST; 2017. Available from: https://tac.nist.gov/publications/2017/additional.papers/TAC2017.ADRoverview.proceedings.pdf.

[16] Xu J, Lee H, Ji Z, Wang J, Wei Q, Xu H. UTH_CCB System for Adverse Drug Reaction Extraction from Drug Labels at TAC-ADR 2017. In: Proceedings of the 2017 Text Analysis Conference. NIST; 2017. Available from: https://tac.nist.gov/publications/2017/participant.papers/TAC2017.UTHCCB.proceedings.pdf.

[17] IBM Research System at TAC 2017: Adverse Drug Reactions Extraction from Drug Labels. In: Proceedings of the 2017 Text Analysis Conference. NIST; 2017. Available from: https://tac.nist.gov/publications/2017/participant.papers/TAC2017.IBMResearch.proceedings.pdf.

[18] Chiu B, Crichton G, Korhonen A, Pyysalo S (2016). How to train good word embeddings for biomedical NLP. Proceedings of BioNLP16.

[19] Hur J, Schuyler AD, States DJ, Feldman EL (2009). SciMiner: web-based literature mining tool for target identification and functional enrichment analysis. Bioinformatics.

[20] Ma X, Hovy E. End-to-end Sequence Labeling via Bi-directional LSTM-CNNs-CRF. In: Proceedings of the 54th Annual Meeting of the Association for Computational Linguistics (Volume 1: Long Papers). Berlin: Association for Computational Linguistics; 2016. p. 1064–74. Available from: https://www.aclweb.org/anthology/P16-1101.

[21] Reimers N, Gurevych I. Reporting Score Distributions Makes a Difference: Performance Study of LSTM-networks for Sequence Tagging. In: Proceedings of the 2017 Conference on Empirical Methods in Natural Language Processing. Copenhagen, Denmark: Association for Computational Linguistics; 2017. p. 338–48. Available from: https://www.aclweb.org/anthology/D17-1035.

[22] Mikolov T, Sutskever I, Chen K, Corrado GS, Dean J. Distributed representations of words and phrases and their compositionality. In: Advances in neural information processing systems; 2013. p. 3111–9.

[23] Pyysalo S, Ginter F, Moen H, Salakoski T, Ananiadou S (2013). Distributional Semantics Resources for Biomedical Text Processing.

[24] Hochreiter S, Schmidhuber J (1997). Long short-term memory. Neural Comput.

[25] Bengio Y, Simard P, Frasconi P (1994). Learning long-term dependencies with gradient descent is difficult. IEEE.

[26] Pascanu R, Mikolov T, Bengio Y (2013). On the difficulty of training recurrent neural networks: international conference on machine learning.

[27] Lample G, Ballesteros M, Subramanian S, Kawakami K, Dyer C. Neural Architectures for Named Entity Recognition. In: Proceedings of the 2016 Conference of the North American Chapter of the Association for Computational Linguistics: Human Language Technologies. San Diego: Association for Computational Linguistics; 2016. p. 260–70. Available from: https://www.aclweb.org/anthology/N16-1030.

[28] Srivastava N, Hinton G, Krizhevsky A, Sutskever I, Salakhutdinov R. Dropout: a simple way to prevent neural networks from overfitting. J Mach Learn Res. 2014;15(1):1929–1958.

[29] Gal Y, Ghahramani Z. A theoretically grounded application of dropout in recurrent neural networks. In: Advances in neural information processing systems; 2016. p. 1019–27

[30] Lafferty J, McCallum A, Pereira FC (2001). Conditional random fields: probabilistic models for segmenting and labeling sequence data.

[31] Dozat T (2016). Incorporating nesterov momentum into adam.

[32] Hur J, Özgür A, Xiang Z, He Y (2015). Development and application of an interaction network ontology for literature mining of vaccine-associated gene-gene interactions. J Biomed Semantics.

[33] Hur J, Xiang Z, Feldman EL, He Y (2011). Ontology-based Brucella vaccine literature indexing and systematic analysis of gene-vaccine association network. BMC Immunol.

[34] Hur J, Özgür A, He Y (2017). Ontology-based literature mining of *E. coli* vaccine-associated gene interaction networks. J Biomed Semantics.

[35] Hur J, Özgür A, He Y (2018). Ontology-based literature mining and class effect analysis of adverse drug reactions associated with neuropathy-inducing drugs. J Biomed Semantics.

[36] Demner-Fusman D, Shooshan SE, Rodriguez L, Aronson AR, Lang F, Rogers W (2018). A dataset of 200 structured product labels annotated for adverse drug reactions. Scientific Data.

